# Vaccination history in elementary school children enrolled in the varicella epidemic investigations held in Jeju-si, Korea in the first half of 2017

**DOI:** 10.4178/epih.e2017053

**Published:** 2017-11-13

**Authors:** Hyun-Suk Oh, Jong-Myon Bae

**Affiliations:** 1 Jeju Heathcare Center of Jeju-si, Jeju, Korea; 2 Department of Preventive Medicine, Jeju National University Scool of Medicine, Jeju, Korea

**Keywords:** Varicella, Vaccination, Chickenpox vaccine, Immunization, Epidemics

## Abstract

**OBJECTIVES:**

The reported incidence rate of varicella infection in Jeju-do is higher compared with the national average. This study aimed to examine varicella vaccination history and evaluate clinical manifestation of varicella cases in Jeju-do.

**METHODS:**

Based on the guideline suggested by the Korea Centers for Disease Control and Prevention (KCDC), two epidemic investigations for varicella infection were conducted in the first half of 2017. The history of varicella vaccination was confirmed using the Integrated Control System for Diseases and Health operated by the KCDC.

**RESULTS:**

Out of a total of 60 elementary school children as the study subjects, all had been previously vaccinated against varicella. Twenty cases (33%) showed mild clinical manifestations and no complications.

**CONCLUSIONS:**

As the government of Jeju-do has supplied a single-labeled vaccine since 2011, there is a need to evaluate the type of vaccination failure such as primary or secondary.

## INTRODUCTION

The reported incidence rate of varicella between 2001 to 2016 in Jeju-do, Korea was about 2.5 times higher than the national average [1]. It was necessary to investigate whether the higher incidence rate was associated with the difference in vaccination rates between the province city and the nation as a whole [2]. According to the Korean Statistical Information Service (http://kosis.kr), the 2016 varicella vaccine coverage was 97.0% in Jeju-do, which is similar to the national average of 97.5% [3]. Since 2011, Jeju-do health authorities have provided free varicella vaccines for children aged 12 through 15 months. The higher incidence of varicella is questionable, thereby requiring further investigation.

On the other hand, while the effectiveness of the varicella vaccine has been questioned in Korea [4,5], it is known that breakthrough varicella infection could occur in children previously vaccinated [6,7]. This study, therefore, aimed to investigate vaccine status among school-age children with varicella in Jeju-do and whether they developed infectious breakthrough varicella despite vaccination. We also aimed to establish research hypotheses that could explain the reportedly high incidence of varicella infection in Jeju-do.

## MATERIALS AND METHODS

According to the Korea Centers for Disease Control and Prevention (KCDC) guidelines [8], an epidemic investigation is required if varicella outbreaks including index cases occur in more than 5% of students in a classroom within a 3-week period in schools, kindergartens and day care centers. In compliance with the guidelines, two varicella outbreak investigations were conducted by the public health centers in two elementary schools in Jeju-si during the first half of 2017. More than 5% of 30 students developed varicella in each of the two classrooms (1st and 3rd grades) (Table 1).

Data on varicella cases were collected using the report from provided by the KCDC for epidemic investigation of varicella including index cases [8]. The vaccination records of the students who were enrolled in the study were checked via the Integrated Diseases and Health Control System of the KCDC (https://is.cdc.go.kr).

## RESULTS

The major findings of the epidemic investigations are summarized in Table 1. Out of 30 students in each class, the number of students with confirmed or suspected varicella was 9 (30.0%) and 11 (36.7%), respectively. It was also found that all the students in the two classes were previously vaccinated. Regarding clinical symptoms, rash only occurred in 5 (55.5%) and 6 (54.5%) students, respectively, accounting for the majority of varicella cases. The number of blisters < 250 was found in 8 (88.9%) and 10 (90.9%) students, respectively. There were no reported complications. Therefore, most cases showed manifestations of breakthrough varicella.

## DISCUSSION

The results revealed that the students who developed varicella in Jeju-si underwent varicella vaccination at 12 through 15 months of age and that they showed clinical manifestations of breakthrough varicella. These results are consistent with the national epidemic characteristics of varicella [4,6,7], but they do not suffice to explain the higher reported incidence rate during varicella outbreaks in Jeju-do, compared with the national average.

This does not rule out the possibility that the said reportedly higher rate reflects other reasons than an actual increase in varicella incidence. Gregg [9] asserted that a high incidence of infectious diseases may result from various factors such as changes in the reporting system, increased awareness about the concerned disease and medical facilities, and changes in diagnostic techniques, in addition to the actual increase in cases due to outbreaks. The last reason regarding changes in diagnostic techniques can be ruled out because most varicella cases are usually identified as suspected varicella. Based on the remaining three reasons, we hypothesized that the number of cases of varicella reported from newly opened pediatric hospitals was higher, when compared with the average reporting rate of other cities, to explain the higher reported rate of varicella incidence in Jeju-do. If this hypothesis is true, it could be concluded that the incidence rate of varicella in Jeju-do reflects the national incidence of varicella and that the pre-existing national average is underestimated.

However, caution is needed when interpreting the results of this study because confirmatory tests were not performed for index cases although this is required by the KCDC guidelines [8]. Under the guidelines, when varicella infection is suspected in the presence of an epidemiological link (a classroom setting) and related clinical symptoms (including blisters), immediate reporting is required and specimens from more than 10% of the patients with suspected varicella or at least two of them should be collected to confirm. To ensure an epidemiological linkage on the site, the specimens of the index cases in which clinical symptoms were initially discovered are primarily collected. In this study, varicella-zoster virus was detected from swab specimens collected from intraoral blisters of those selected as primary candidates. The remaining subjects were categorized as the suspected cases, which should be remembered when interpreting the results.

Based on the report that seropositivity is reduced to 40% at the age of 4 years [10] and varicella control practices in the US [11], the need for two doses of vaccination can be considered in Korea [12]. However, as long as there is uncertainty regarding vaccine effectiveness [4,5], two-doses of vaccination could be meaningless. Moreover, the incidence of breakthrough varicella in vaccinated children underlines that further studies are needed to ascertain whether it stems from primary vaccine failure where vaccine fails to provide immunity or secondary vaccine failure where immunity is lost over time [13]. Considering that free vaccines have been provided in Jeju-do since 2011 using a single type of varicella vaccine, this study suggests the use of different vaccines of other pharmaceutical companies and subsequently a community trial to track the changes in the reported incidence rate of varicella infection. The results of continued studies will be useful to determine future prevention and control policies for varicella including the decision for vaccine replacement or two-doses of vaccination.

## Figures and Tables

**Table 1.** Summary of two epidemiologic investigations carried out in Jeju-si, Korea in the first half of 2017

School	Elementary school A	Elementary school B	Total
Study period	Apr 12-Apr 26	May 10 - Jun 16	
Grade	1st	3rd	
No.of children in a class	30	30	60
Incidence (%) in the class	30.0% (9/30)	36.7% (11/30)	33.3% (20/60)
History of vaccination (%)	100	100	100
Clinical manifestation (n, column %)	9	11	20
Rash only	5 (55.6)	6 (54.5)	11 (55.0)
Rash + fever	3 (33.3)	3 (27.3)	6 (30.0)
Rash + fever + headache	1 (11.1)	2 (18.2)	3 (15.0)
No. of vesicles (n, column %)	9	11	20
≤49	2 (22.2)	4 (36.4)	6 (30.0)
50-249	6 (66.7)	6 (54.5)	12 (60.0)
≥250	1 (11.1)	1 (9.1)	2 (10.0)
