# Supplementary material for: Vaccination history in elementary school children enrolled in the varicella epidemic investigations held in Jeju-si, Korea in the first half of 2017
Source: Epidemiol Health. 2017 Nov 13;39:e2017053. doi: 10.4178/epih.e2017053 (PMC5790978; doi:10.4178/epih.e2017053)
Supplement: Supplementary file 1 [file epih-39-e2017053-supplementary.pdf]

Volume: 39, Article ID: e2017053
https://doi.org/10.4178/epih.e2017053

<Epidemiologic Investigation>

2017년도 상반기 제주시 수두 역학조사 대상자의 수두백신 접종력

Vaccination history in elementary school children enrolled in the varicella epidemic investigations held in Jeju-shi, Korea in the first half of 2017

Hyun-Suk Oh[1], Jong-Myon Bae[2]

[1]Jeju Heathcare Center of Jeju-si, Jeju, Korea; [2]Department of Preventive Medicine, Jeju National University Scool of Medicine, Jeju, Korea

Correspondence: Jong-Myon Bae

Department of Preventive Medicine, Jeju National University School of Medicine

102 Jejudaehak-ro, Jeju 63243, Korea

E-mail: jmbae@cheju.ac.kr

Volume: 39, Article ID: e2017053
https://doi.org/10.4178/epih.e2017053

Abstract

Objectives: The reported incidence rate of varicella infection in Jeju-do is higher compared with the national average. This study aimed to examine varicella vaccination history and evaluate clinical manifestation of varicella cases in Jeju-do.

Methods: Based on the guideline suggested by Korea Centers for Disease Control and Prevention (KCDC), two epidemic investigations for varicella infection were conducted in the first half of 2017. The history of varicella vaccination was confirmed using the Integrated Control System for Diseases and Health operated by the KCDC.

Results: Out of a total of 60 elementary school children as the study subjects, all had been previously vaccinated against varicella. Twenty cases (33%) showed mild clinical manifestations and no complications.

Conclusions: As the government of Jeju-do has supplied a single-labeled vaccine since 2011, there is a need to evaluate the type of vaccination failure such as primary or secondary.

Keyword: Varicella, Vaccination, Chickenpox vaccine, Immunization, Epidemics

Volume: 39, Article ID: e2017053
https://doi.org/10.4178/epih.e2017053

서론

최근에 제주도의 2001-2016년도 수두 발생 신고률이 전국에 비해 약 2.5배 더 높다는 보고가 있었다 [1]. 이렇게 전국에 비해 높은 발생률을 보인 것에 대하여 수두 백신 접종률의 차이가 있는가를 우선 확인해 봐야 한다 [2]. 이에 국가통계포털 (Koran Statistical Information Service, http://kosis.kr)에서 2016년도 수두백신 접종률을 살펴보았을 때 전국 97.5%에 비하여 제주도는 97.0%로 전국과 차이가 없었다 [3]. 제주도는 2011년부터 무상으로 12-15개월 영아에 대하여 무상으로 접종을 시행하고 있기에, 왜 제주도의 수두 발생률이 전국에 비하여 높은 가에 대하여 추가적인 연구가 필요하다.

한편 국내 수두 백신의 효과 (effectiveness)에 대하여 의문이 제기되었으며 [4,5], 접종력이 있는 아이에서 돌발수두 (breakthrough varicella)가 발생한다고 보고하였다 [6,7]. 이에 본 연구의 목적은 제주도 수두 발생자들에서 백신 접종력 여부와 함께, 접종력이 있음에도 감염증상을 보이는 돌발수두 발생 여부를 알아보는 것이다. 이를 통해 제주도 수두 발생 신고율이 높은 것을 설명하는 연구 가설을 도출할 것을 기대한다.

Volume: 39, Article ID: e2017053
https://doi.org/10.4178/epih.e2017053

연구대상 및 방법

질병관리본부의 지침 [8]에 따르면 학교, 유치원, 영유아 보육시설 등에서 3주 이내 1개 학급에서 수두(의사) 환자가 해당 학급 전체 인원의 5% 이상 발생한 경우에 역학조사를 수행하도록 되어 있다. 이상의 지침에 따라 2017년 상반기 중에 제주도 제주시 제주보건소 담당 관내 초등학교 2 곳에서 유행조사를 시행하였다. 1학년과 3학년 학급에서 학생수 각각 30명으로, 5% 이상의 환자가 발생하였기 때문이다 (Table 1).

질병관리본부가 제시한 수두 (의사)환자 역학조사서 [8]에 따라 관련 정보를 수집하였으며, 조사대상 학생의 수두접종력은 질병관리본부가 운영 및 관리하는 '질병보건통합관리시스템' (https://is.cdc.go.kr)를 이용하여 확인하였다.

연구결과

역학조사의 주요 결과를 표 1로 요약하였다. 두 학급 학생 각 30명 중 의사환자를 포함한 수두 환자 발생은 각각 9명 (30%), 11명 (36%)이었다. 환자뿐만 아니라 발생하지 않은 학급 학생 전부가 수두 접종력이 있음을 확인하였다. 임상증상으로 발진만 나타난 경우가 5명 (55.5%), 6명 (54.5%)로 과반수를 넘었고, 물집이 250개 미만이 8명 (88.9%), 10명 (90.9%)이고, 합병증을 가진 사례는 전무하여 대부분 돌발수두 양상을 보였다.

Volume: 39, Article ID: e2017053
https://doi.org/10.4178/epih.e2017053

토론

이상의 결과들에 따르면, 제주시 수두 발생자들은 모두 생후 12-15개월에 수두 백신 접종력을 가지고 있으며, 돌발수두의 임상증상을 보이는 것으로 요약된다. 이는 전국적인 수두 발생의 역학적 특성과 일치하는 것일 뿐 [4,6,7], 제주도가 전국에 비하여 높은 발생 신고율을 보이는 것에 대한 설명으로는 부족하다.

그렇다면, 제주도에서 수두 발생률이 높은 것은 실제 발생의 증가라기보다는 다른 이유를 고려해야 한다. Gregg [9]는 특정 감염병의 발생률이 높은 배경에는 실제 발생의 증가 이외에도, 신고체계의 변화, 해당 질병에 대한 인지도 증가, 새로운 의료기관의 설립, 진단방법의 변화 등이 있다고 지적하였다. 이중 마지막 이유인 진단방법의 변화는 배제할 수 있는데, 대부분의 신고가 의사환자로 이루어 지기 때문이다. 그렇다면 나머지 3가지 요소들을 묶어서 제주도내 개원 소아과 의원의 신고율이 타 시도에 비하여 높다는 가설 제시는 제주도의 수두 발생 신고율이 전국에 비하여 높은 현상을 설명할 수 있다. 만약 이 가설이 맞다면, 제주도의 수두발생 수준이 전국적인 실제 발생수준을 의미하며, 현재의 전국 발생률은 저평가되었다고 해석할 수 있다.

Volume: 39, Article ID: e2017053
https://doi.org/10.4178/epih.e2017053

본 연구결과에 있어 해석상 주의를 요하는 것은 질병관리본부의 역학조사지침 [8]에 따라, 수두로 의심되는 환아 모두에 대하여 확진검사를 시행하지 않았다는 점이다. 지침에 따르면, 진단을 위한 검사기준에 부합하는 검사결과가 없지만 역학적 연관성 (한 학급내)과 수두에 부합하는 임상증상 (수포 등)을 보인 '의사환자'는 지체없이 신고를 하게 되어 있으며, 총 의심환자의 10% 이상 혹은 최소 2 명 이상에서 확진을 위해 검체를 채취하도록 되어 있다. 현장에서는 역학적 연관성을 확보하기 위하여 임상증상을 최초로 보인 의사환자 (index case)를 우선 채취 대상자로 선정한다. 이번 연구에서는 선정된 환아 모두의 구강내 수포에서 채취한 인두도찰물에서 수두 바이러스 검출이 되었다. 나머지 환아는 의사환자로 조사되었다는 점을 해석상 유의할 점이다.

혈청학적 양성률 (seropositivity)이 4세가 되면 40%로 감소한다는 보고 [10]와 함께 미국의 수두 관리 경험 [11]에 따라, 우리나라도 2번 접종을 할 필요성을 제기할 수 있겠다 [12]. 그러나 현재 국내에서 접종하는 백신의 예방 효과에 대한 의문이 있는 상황 [4,5]에서는 2회 접종은 무의미할 수 있다. 더군다나 접종력이 있는 환아에서의 돌발수두가 접종 후 면역력 획득이 안된 1차 백신 실패 (primary vaccine failure)인가, 아니면 접종 이후 시간이 경과해서 면역력이 떨어진 2차 백신실패 (secondary vaccine failure)인가에 대하여도 추가 연구가 필요하다 [13]. 현재로서는 제주도는 2011년 무상접종사업을 펼친 이후 현재까지 동일 회사의 동일 백신을 접종해왔다는 점에서, 향후에 다른 제약사의 백신을 공급하여 접종을 하여서 수두 발생신고율의 변화 추이를 살펴보는 지역사회 시험 (community trial) 을 수

행하는 것을 제안한다. 그 결과에 따라 현재의 백신을 다른 것으로 바꿀 것인지, 아니면 2회 접종으로 갈 것인지의 수두예방 관리 정책을 결정할 수 있기 때문이다.

Acknowledgement

본 연구는 2017년 지자체 역량강화 실무자교육 과정 (Field Management Training Program)을 통해 연구가 수행되었음을 밝힙니다.

References

1. Park JE, Kim SY, Yang DH, Bae JM. Status of national notifiable infectious disease in Jejudo, Korea: 2001-2016. J Med Life Sci 2017;8(1):29-34. (korean)

2. Vázquez M, LaRussa PS, Gershon AA, Steinberg SP, Freudigman K, Shapiro ED. The effectiveness of the varicella vaccine in clinical practice. N Engl J Med 2001;344(13):955-60.

3. Korean Statistical Information Service. National vaccination rates by sex.[cited 2017 Oct 21]. Available from: http://kosis.kr/statHtml/statHtml.do?orgId=117&tblId=DT_11793N_152&vw_cd=MT_ZTITLE&list_id=D1_A01_A02&seqNo=&lang_mode=ko&language=kor&obj_var_id=&itm_id=&conn_path=E1

4. Oh SH, Choi EH, Shin SH, Kim YK, Chang JK, Choi KM, et al. Varicella and varicella vaccination in South Korea. Clin Vaccine Immunol 2014;21(5):762-8.

5. Lee YH, Choe YJ, Cho SI, Kang CR, Bang JH, Oh MD, et al. Effectiveness of varicella vaccination program in preventing laboratory-confirmed cases in children in Seoul, Korea. J Korean Med Sci 2016;31:1897-1901,

6. Park CS, Kim DS, Kim KH. Varicella outbreak in the patients during group therapy: seroprevalence in a healthcare system during breakthrough varicella occurrence. Clin Exp Vaccine Res 2013;2:140-143.

7. Park CK, Kim HO,Park CW, Lee CH. The effect of active immunization on the clinical courses of varicella. Korean J Dermatol 2008;46(8):1011-1019. (korean)

8. Korea Center for Diseases Control and Prevention. Guidelines for Vaccine-controllable infectious diseases in 2016. [cited 2017 Oct 21]. Available from: http://cdc.go.kr/CDC/together/CdcKrTogether0302.jsp?menuIds=HOME001-MNU1154-MNU0005-MNU0088&fid=51&q_type=&q_value=&cid=74163&pageNum=1

9. Michael Gregg. Field Epidemiology 3rd ed. Oxford University Press; 2008. p.85.

10. Choi UY, Huh DH, Kim JH, Kang JH. Seropositivity of Varicella zoster virus in vaccinated Korean children and MAV vaccine group. Hum Vaccin Immunother 2016;12(10):2560-2564.

11. Lopez AS, Zhang J, Marin M. Epidemiology of Varicella During the 2-Dose Varicella Vaccination Program - United States, 2005-2014. MMWR Morb Mortal Wkly Rep 2016;65(34):902-905.

12. Perella D, Wang C, Civen R, Viner K, Kuguru K, Daskalaki I, et al. Varicella Vaccine Effectiveness in Preventing Community Transmission in the 2-Dose Era. Pediatrics 2016;137(4). pii: e20152802.

13. Papaloukas O, Giannouli G, Papaevangelou V. Successes and challenges in varicella vaccine. Ther Adv Vaccines 2014;2(2):39-55.

Volume: 39, Article ID: e2017053
https://doi.org/10.4178/epih.e2017053
